# Insulin treatment prevents wounding associated changes in tissue and circulating neutrophil MMP-9 and NGAL in diabetic rats

**DOI:** 10.1371/journal.pone.0170951

**Published:** 2017-02-09

**Authors:** Maryam Abdollahi, Taria Shin Yi Ng, Alireza Rezaeizadeh, Sarah Aamidor, Stephen M. Twigg, Danqing Min, Susan V. McLennan

**Affiliations:** 1 Greg Brown Diabetes and Endocrinology Laboratories, Sydney Medical School, Charles Perkins Centre, University of Sydney, Sydney, New South Wales, Australia; 2 Department of Endocrinology and Diabetes Centre, Royal Prince Alfred Hospital, Sydney, New South Wales, Australia; 3 Chemical Pathology, Royal Prince Alfred Hospital, NSW Health Pathology, Sydney, New South Wales, Australia; University of Edinburgh, UNITED KINGDOM

## Abstract

Neutrophils are important for wound repair, but their persistence can impair the healing process. Neutrophils express matrix metalloproteinases including MMP-9 and its regulator neutrophil gelatinase associated lipocalin (NGAL). Whether wounding affects neutrophil MMP-9 and NGAL in diabetic animals is not known. Skin wound tissue MMP-9 and NGAL was examined by qRT-PCR and immunohistochemistry in control, diabetic and insulin treated diabetic rats. The temporal expression of MMP-9 and NGAL mRNA, MMP-9 activity and the NGAL/MMP-9 complex was also investigated in an implant model and their circulating neutrophils. The cellular localisation of MMP-9 and NGAL was confirmed by immunofluorescence and the ability of glucose to regulate these factors was examined in isolated neutrophils. In skin wound tissue compared with control, diabetes increased neutrophil infiltration, NGAL mRNA and MMP-9 protein (P<0.05). Diabetes significantly increased implant neutrophil NGAL and MMP-9 protein as well as NGAL mRNA, wound fluid NGAL/MMP-9 complex and MMP-9 activity (all <0.05). Circulating neutrophil MMP-9 and NGAL was also increased in these diabetic animals (P<0.05). These changes were prevented by insulin treatment. Ex vivo, high glucose (25mM) increased neutrophil NGAL and MMP-9 (both by 2 fold, P<0.05). NGAL and MMP-9 are increased in wound and circulating neutrophils in diabetic rodents. These changes and the association between higher NGAL and increased wound fluid MMP-9 activity suggest that increased neutrophil NGAL may contribute to increased MMP-9 in poorly healing diabetic wounds. Whether targeting neutrophil NGAL or MMP-9 can improve diabetic wound healing remains to be investigated.

## Introduction

Delayed healing of wounds is a poorly understood complication that affects 15–30% of all persons with diabetes [1, 2]. It is also associated with much morbidity and the escalating number of people with diabetes in an ageing population will increasingly add to the economic and human burden of this diabetic complication. For these reasons understanding the pathophysiology of poor wound healing in diabetes is of critical importance.

The normal wound healing process is complex and composed of at least three distinct but overlapping stages including inflammation, proliferation and remodelling. Neutrophils are essential for healing; they are the first responders and act to scavenge debris and deliver chemokines to recruit monocytes and lymphocytes to the inflammatory site. In poorly healing or chronic wounds neutrophils and macrophages persist and accumulate and pro-inflammatory cytokines and proteases are increased [3, 4]. Neutrophils are a rich source of proteolytic enzymes including matrix metalloproteinases (MMPs), in particular MMP-9 and MMP-8 which are secreted by neutrophils on their activation and degranulation [5–7]. Increased MMP levels are associated with poor wound healing [8]. In chronic wounds MMP-9 is increased in wound tissue as well as wound fluids [9, 10] and elevated tissue levels of MMP-9 are associated with poor wound healing in diabetes [10]. We have previously shown that increased wound fluid MMP-9 can predict future delayed healing of neuropathic diabetic foot ulcers [11].

MMP expression and activity is tightly regulated at multiple levels [12]. Unlike most cells in which MMP-9 activities are regulated by tissue inhibitors of metalloproteinases (TIMPs), neutrophils are relatively TIMP free [13, 14]. In addition to regulation by TIMPs, MMP-9 activity can be regulated by neutrophil gelatinase-associated lipocalin (NGAL). NGAL also known as lipocalin 2, is a 25 kDa secreted protein that is involved in the allosteric activation of MMP-9. It can also form complex with MMP-9 that stabilizes MMP-9 and protects it from degradation [15]. NGAL is stored in the secondary granules of neutrophils and like MMP-9 is secreted during neutrophil activation and degranulation [16]. In addition to regulation of MMP-9, NGAL has a high affinity for siderophores and is involved in the neutrophil response to infection, suggesting a role in the innate immune system. NGAL is also important for cell homeostasis as well as tissue differentiation and repair [16].

Despite these known roles, little is known regarding the role of NGAL in wound healing. Additionally the effect of diabetes on NGAL and MMP-9 levels in wound and in circulating neutrophils has received little attention. In the present study we used an excisional wound model to examine NGAL and MMP-9 levels in wound tissue. An implant wound model was used for more detailed examination of the temporal expression of NGAL and MMP-9 in neutrophils and wound fluids. The effect of diabetes on circulating neutrophil MMP-9 and NGAL and the effect of insulin treatment was also studied.

## Materials and methods

### Animal studies

This study was approved by the Animal Ethics Committee of Sydney South West Area Health Service. All experiments were conducted in accordance with the Principles of Laboratory Animal Care (NIH Publication no. 85–23, revised 1985) and National laws. Male Sprague-Dawley rats, aged between 5 and 6 weeks were obtained from the Animal Resources Centre (Perth, Australia). Diabetes was induced by a single intraperitoneal injection of streptozotocin (STZ; 65mg/kg in 0.1M citrate buffer, pH4.5, Calbiochem) and confirmed by tail vein blood glucose level > 11mmol/L. One week later the diabetic animals were randomly divided into two groups, a maintenance group receiving a low dose insulin regimen (DM: 2-4IU Mixtard insulin, twice weekly, n = 35) to maintain body weight and prevent ketoacidosis, and an intensively treated group (DM+INS, n = 20) who received daily insulin at a dose of 10IU/day, until 12 hours prior to termination. Non-diabetic age matched rats acted as control (CON, n = 25). Six weeks later, the animals were anaesthetized using a combination of Ketamine (85mg/kg) and Xylazine-20 (5mg/kg) the dorsum was shaved and the hair removed using Veet (Reckitt Benckiser, Berkshire, UK). The dorsum was then swabbed with Betadine (Sanofic, Netherlands) and poly vinyl alcohol (PVA) sponges sterilised by gamma irradiation (1cm^2^, 4 per animal) were implanted via 4 x 1cm incisions on the dorsum of the animal. The incisions were then closed with suture. At the time of surgery each animal received antibiotics (Ampicillin; 50mg/kg) and Buprenorphine (Temgesic 0.03 mg/kg) for pain relief. After 3, 6, and 12 days the animals were anaesthetized with ketamine, blood was collected via cardiac puncture and the animals were euthanized by exsanguination. The dorsum was then swabbed with 70% ethanol and the implants were removed and placed into sterile containers. The cellular and fluid components contained within the implant were then squeezed from the implant and the cellular and liquid fractions were separated by centrifugation (800g for 10mins). This liquid (termed wound fluid) was stored at -80°C for later analysis. The cell fraction was incubated in red blood cell (RBC) lysis buffer (NH_4_Cl; 155mM, KHCO_3_; 10 mM, EDTA; 100 μM) and washed. The pellet containing the white cells was divided and aliquots were either resuspended in Tri-reagent for later RNA extraction or spun onto slides for examination of neutrophils and macrophages by immunofluorescence.

The effect of diabetes on granulation tissue NGAL and MMP-9 was also studied in a skin excisional wound model. For these studies, the rats were anaesthetized and the dorsum was prepared for wounding as described above. Four full-thickness circular wounds (8mm^2^) were then created on the dorsum using a biopsy punch as previously described[17]. Wound area was traced daily for determination of wound healing rate (calculated as change in wound area /day) and at day 6 post wounding the animals (n = 5-6/group) were euthanized and the skin containing the wound tissue was excised. Two wounds were snap frozen in liquid N_2_ for later measurement of gene expression and the other wounds were divided in half and fixed in formalin (10%) for histological and immunohistological studies or frozen in OCT for immunofluorescence staining.

### Isolation of neutrophils

Whole blood collected from animals in the implant study at euthanasia was used for measurement of plasma and neutrophil NGAL and MMP-9. The heparinised blood was centrifuged (800g, 15mins) and the plasma was removed and stored. The packed cells were resuspended in sterile Hanks’ balanced salt solution (HBSS; 1:1 vol/vol) and the solution was overlayed on Histopaque-1083 and Dextran as previously described to isolate neutrophils [18]. Neutrophil aliquots were stored in Tri-reagent for RNA extraction, or spun onto slides for histological and immunofluorescence studies. The purity of the preparation was assessed using May-Grunwald-Giemsa stain and was routinely greater than 95%.

To examine the direct effect of glucose on neutrophil MMP and NGAL expression, neutrophils isolated from whole blood obtained from control unwounded rats were cultured in a humidified atmosphere of 5% CO_2_ in air at 37°C in RPMI media containing 5 or 25mM glucose for 2 hours. The mRNA levels of NGAL, MMP-9, and MMP-8 as well as TLR4, TLR2, and TNFα, as pro-inflammatory mediators, and the anti-apoptosis marker Mcl-1 were measured by qRT-PCR.

### Immunofluorescence and immunohistochemistry

Implant neutrophil and macrophage numbers were determined by staining for Myeloperoxidase (MPO; ab90810, Abcam) and CD68 (ab31630, Abcam) respectively. Cellular localisation of NGAL and MMP-9 in implant inflammatory cells and isolated peripheral blood neutrophils was determined using antiNGAL (M145, SantaCruz) and antiMMP-9 (sc-6840, SantaCruz) antibodies according to standard procedures [19]. The secondary antibodies (Alexa Fluor 488 or 594, Invitrogen) were used for visualisation and the nuclei were stained with DAPI. Images were captured using an Olympus AX-70-Fluorescence microscope.

The cellular localisation of NGAL (ab41105; Abcam) and MMP-9 (ab76003; Abcam) was examined in skin wound tissue by immunohistochemistry. For quantitation of NGAL, the staining intensity was examined in the epithelial (top), granulation tissue (middle) and inflammatory (basal) zones and scored by two independent observers based on a scale of 0 = no staining above isotype control to 3 = intense staining. The score for each zone was summed and the data was analysed using Chi squared analysis at an intensity cut off at ≥ 6. MMP-9 staining was localised mainly to cells in the granulation tissue layer and the staining intensity in this area was quantified using Image J software.

### Flow cytometry studies

The effect of diabetes on peripheral blood neutrophil number was determined in blood obtained at euthanasia from animals used in the implant study. Flow cytometry (FACS Canto Flow Cytometer) analysis was based on morphology using forward scatter (FSC) and side scatter (SSC) and the data was analysed using FlowJo software (Treestar FlowJo v X10).

### RNA isolation and quantitative RT-PCR

Total RNA was isolated from the implant cellular infiltrate, skin excisional wound tissue or peripheral blood neutrophils using TriReagent (Invitrogen). The RNA concentration and purity of each sample was determined using Nanodrop (BioRad). RNA (1μg) was reverse transcribed to cDNA using Oligo(dT)_18_ primers and Superscript III Reverse Transcriptase (Invitrogen). For analysis all mRNAs were amplified in duplicate using a Rotorgene 6000 (Corbett), SensiMix SYBR Hi-ROX kit (Bioline) and primers specific for NGAL, MMP-9 and MMP-8. The expression of TNFα and TLR4 and TLR2 were measured as markers of inflammation. The single copy gene *36b4* was used as the reference gene. The primer sequences are shown in Table A in S1 File. All PCR products amplified in parallel with *36b4*, the no template control (NTC) failed to amplify and melt curve analysis showed a unique peak for all primer pairs (not shown). The data was analysed using the delta/delta method and expressed as fold change from control.

### Gelatin zymography

The total MMP-9 activity, pro- and active MMP-9 and the NGAL/MMP-9 complex in wound fluids obtained from the PVA implants was determined by MDPF-labeled gelatin zymography [20, 21]. The gel images were digitised using the Bio-Rad ChemiDoc MP Imaging System and the relative intensity of each band was determined by measurement of the peak area (AUC) using Phoretix 1D (Advanced version 3.01). Bands corresponding to NGAL/MMP-9 complex (~115,125 kDa), pro-MMP-9 (~92 kDa) and active-MMP-9 (~82 kDa) were observed and their identity was confirmed by Western blot analysis using antiNGAL (ab63929, Abcam) and antiMMP-9 antibodies (ab38898, Abcam) (data not shown).

### Measurement of NGAL concentration

Wound fluid NGAL concentration was determined using an ELISA that detected only unbound NGAL (R&D Systems).

### Statistical analysis

All data is expressed as mean ± standard error (SEM) and was analysed using the Statistical Package for the Social Science (SPSS for Windows, version 20). Except where mentioned data was analysed using one-way ANOVA followed by Tukey's post-hoc test for multiple comparisons. Statistical significance was accepted at a 95% confidence level when P<0.05.

## Results

### Animal characteristics

As expected the diabetic animals weighed less and had higher non-fasting blood glucose levels (BGL) than control animals. Insulin treatment at 10IU per day partially normalised these parameters with near-normal blood glucose levels recorded at 4 and 12 hours post injection (Table 1).

**Table 1:** Animal characteristics.

Groups	Termination weight (g)	Non fasting BGL (mmol/L)	BGL 4hrs. after insulin injection (mmol/L)	BGL 12 hrs. after insulin injection (mmol/L)
CON	488.4±9.9	5.1±0.1	5.1±0.1	5.0±0.6
DM	373.8±10.3*	21.2±1.4*	19.1±0.8*	21.3±0.8*
DM+INS	421.7±9.7*	17.1±0.6*^#^	3.4±0.4^#^	9.3±1.6*^#^

Results from Control (CON) diabetic (DM) and Insulin treated (DM+INS) animals. Blood glucose levels (BGL) were measured from tail vein blood one week prior to termination. Results are expressed as Mean ± SEM,

*P<0.05 vs. CON,

^#^P<0.05 vs. DM.

### The effect of diabetes on skin wound tissue NGAL and MMP-9

**Fig 1:**
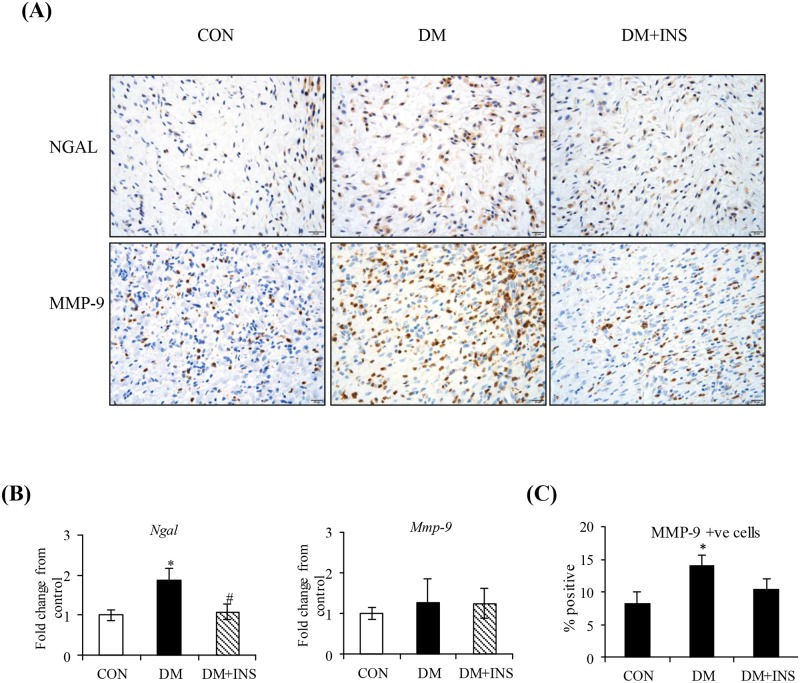
The effect of diabetes on NGAL and MMP-9 in skin wound tissue at day 6. (A) Representative images for skin wound tissue NGAL and MMP-9 respectively (B) Group data for wound NGAL, and MMP-9 mRNA and (C) Group data for percentage MMP-9 staining cells in the granulation tissue. Results are from Control (CON) diabetic (DM) and Insulin treated DM (DM+INS) animals and are expressed as Mean ± SEM. * P<0.05 vs. CON, ^#^P < 0.05 vs. DM.

The effect of diabetes on NGAL and MMP-9 was first examined in skin wound granulation tissue at day 6 post wounding. In this model and similar to our previous studies[17] wound healing rate was decreased in diabetic animals (CON: 1.62 ± 0.09 vs DM: 1.2 ± 0.1 mm^2^/day, P<0.05). Insulin treatment from the onset of diabetes prevented the decrease in wound healing rate (DM+INS 1.34 ± 0.1 mm^2^/day). By immunohistochemistry, NGAL and MMP-9 staining was present in the epidermis, dermis and hypodermis (Figure A in S1 File), with the greatest intensity associated with inflammatory cells in the granulation tissue area of the hypodermis (Fig 1A). The staining intensity of NGAL (Table 2) and the percentage of MMP-9^+ve^ cells (Fig 1C) was significantly (P<0.05) higher in the granulation tissue area of diabetic animals compared with controls. These changes in NGAL but not MMP-9 were also observed at the mRNA level (Fig 1B).

**Table 2:** Scoring of NGAL positive staining in wound tissue.

	Epidermis		Dermis		Hypodermis	
	Scores < 2	Scores ≥ 2	Scores < 2	Scores ≥ 2	Scores < 2	Scores ≥ 2
CON (n = 5)	4	1	5	0	1	4
DM (n = 5)	5	0	1	4	0	5
DM+INS (n = 4)	4	0	4	0	1	3
	Asymp. Sig. (2-sided)					
	CON vs DM			DM vs DM+INS		
Epidermis	0.37			0.27		
Dermis	0.006*,			0.007^#^		
Hypodermis	0.50			0.18		
Overall	0.045*			0.031^#^		

Results from Control (CON) diabetic (DM) and Insulin treated DM (DM+INS) animals are scored and categorized as shown. The data was then analyzed using Chi squared tests and the differences between the CON and DM groups or DM and DM+INS are presented as P values, with P <0.05 being significant.

### The effect of diabetes on PVC implant NGAL and MMP-9 mRNA and protein

**Fig 2:**
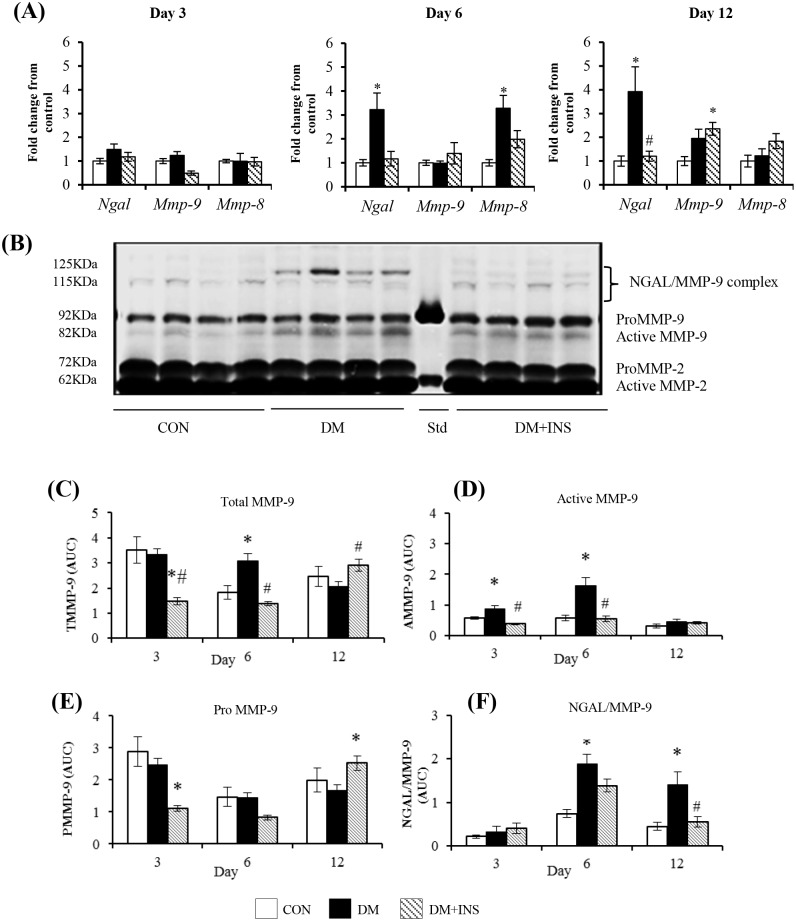
The effect of diabetes on NGAL and MMP-9 expression in PVC implant cells and wound fluids at days 3, 6 and 12. (A) NGAL, MMP-9 and MMP-8 mRNA by qRT-PCR, (B) A representative zymogram of wound fluid at day 6, (C-E) Group data for zymography results expressed as Area Under Curve (AUC; determined from band intensity) (C) total MMP-9 (TMMP-9 = proMMP-9 +active MMP-9), (D) active MMP-9, (E) proMMP-9 and (F) NGAL/MMP-9 complex according to molecular weight. Results are from Control (CON) diabetic (DM) and Insulin treated DM (DM+INS) animals and are expressed as Mean ± SEM *P <0.05 vs. CON, #P <0.05 vs. DM.

To examine in more detail the effect of diabetes on wound inflammatory cells and in particular neutrophil NGAL and MMP-9 we used a PVC implant model. As shown (Fig 2A), no differences were seen in NGAL or MMP-9 mRNA levels at day 3. In contrast, at day 6 in diabetic animals, NGAL mRNA was increased (by 3.5 fold vs control, P<0.05) and this increase persisted at day 12. Diabetes had no effect on MMP-9 mRNA at days 6 or 12 but insulin treatment significantly increased MMP-9 mRNA at day 12 (P<0.05 vs control). MMP-8 mRNA measured at each time point as a neutrophil marker was increased at day 6 but this increase was not apparent at day 12. The day 6 and 12 increases in NGAL mRNA were prevented by insulin treatment.

In PVC implant wound fluids, zymography was used to measure the pro and biologically active forms of MMP-9 and the NGAL/MMP-9 complex (representative zymogram Fig 2B). In both control and diabetic animals Total MMP-9 (TMMP-9:Pro +active forms) was highest at day 3 and decreased with time. This time related decrease in TMMP-9 was not seen in the insulin treated group (Fig 2C). In diabetic animals when compared with control, TMMP-9 was significantly elevated at day 6 (P<0.05). This was due to an increase in the active form of MMP-9 (activeMMP-9, Fig 2D) as in diabetic animals ProMMP-9 was not different to control (Fig 2E). In all animals the NGAL/MMP-9 complex was highest at day 6 and in diabetic animals was significantly higher than control at days 6 and 12 (Fig 2F). Insulin treatment of diabetic animals, decreased Total MMP-9 (both PMMP-9 and AMMP-9) at days 3 and 6 (Fig 2C–2E) and the NGAL/MMP-9 complex at days 6 and 12 (Fig 2F).

By ELISA diabetes decreased wound fluid NGAL concentration (DM: 53.0±9.8 vs. CON: 65.2±4.6), but this failed to reach significance (P<0.06).

### The effect of diabetes on PVC implant neutrophil number and expression of NGAL and MMP-9

**Fig 3:**
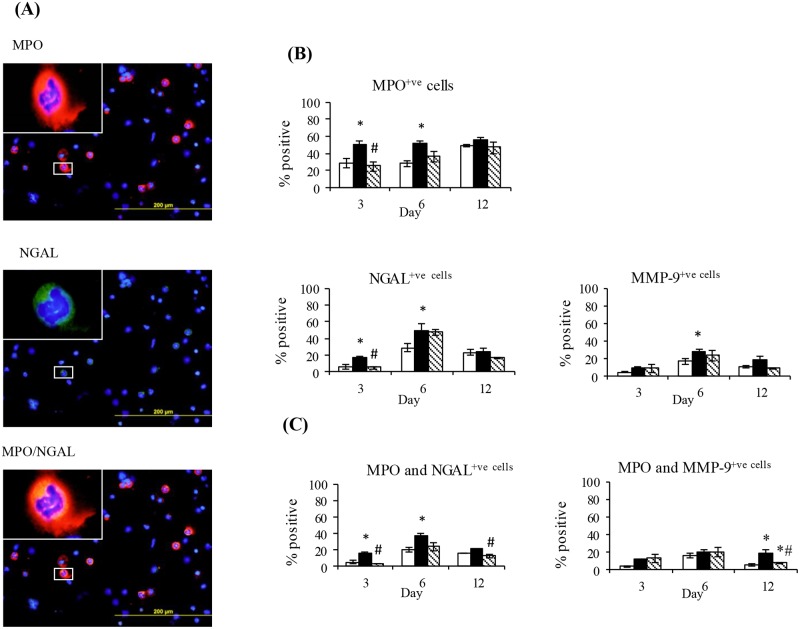
The effect of diabetes on PVC implant cell expression of MPO, NGAL and MMP-9 and the co-localisation of MPO with NGAL or MMP-9. (A) Representative day 6 images of implant cells stained with MPO and NGAL either alone or in combination. Group data for percentage of implant cells expressing (B) MPO, NGAL, or MMP-9 or (C) MPO in combination with either NGAL or MMP-9. Data are from the cellular fraction of implants obtained from Control (CON) Diabetic (DM) and Insulin treated DM (DM+INS) animals at days 3, 6 and 12 post implantation. Results are expressed as Mean ± SEM. * P<0.05 vs. CON, ^#^P <0.05 vs. DM.

The effect of diabetes on implant neutrophils was then examined by immunofluorescence. The cellular infiltrate contained both neutrophils and macrophages at each of these time points. Shown in Fig 3A are representative images of neutrophils stained with MPO (red) and NGAL (green). In control animals the percentage of neutrophils increased with time reaching 42±5% of the cellular infiltrate at day 12 (Fig 3B). Compared to control the percentage of neutrophils was higher in diabetic animals (each by approximately 2 fold, P<0.05) at days 3 and 6. In diabetic animals compared with controls, the percentage of cells expressing NGAL was significantly increased at days 3 and 6 but the percentage of cells expressing MMP-9 was only significantly increased at day 6 (Fig 3B). At day 3 but not days 6 and 12, insulin treatment abolished the increase in MPO positive cells as well as the percentage expressing NGAL (Fig 3B). Co-localisation studies showed in each group at day 3 and 6 approximately 50% of MPO positive cells were also NGAL positive. This percentage decreased at day 12 and was not different across the groups (Fig 3C). At day 6 the percentage of neutrophils expressing MMP-9 was increased in diabetic animals and like the pattern for NGAL was unaffected by insulin treatment (Fig 3C). Interestingly in all groups the majority of MMP-9 positive cells were neutrophils. Shown in Figure B in S1 File at day 6 the percentage of macrophages (CD68 positive) was not altered by diabetes. A small percentage of macrophages were shown to express NGAL and this was increased by diabetes and prevented by insulin treatment.

### The effect of diabetes on circulating neutrophil number and NGAL and MMP-9 expression

**Fig 4:**
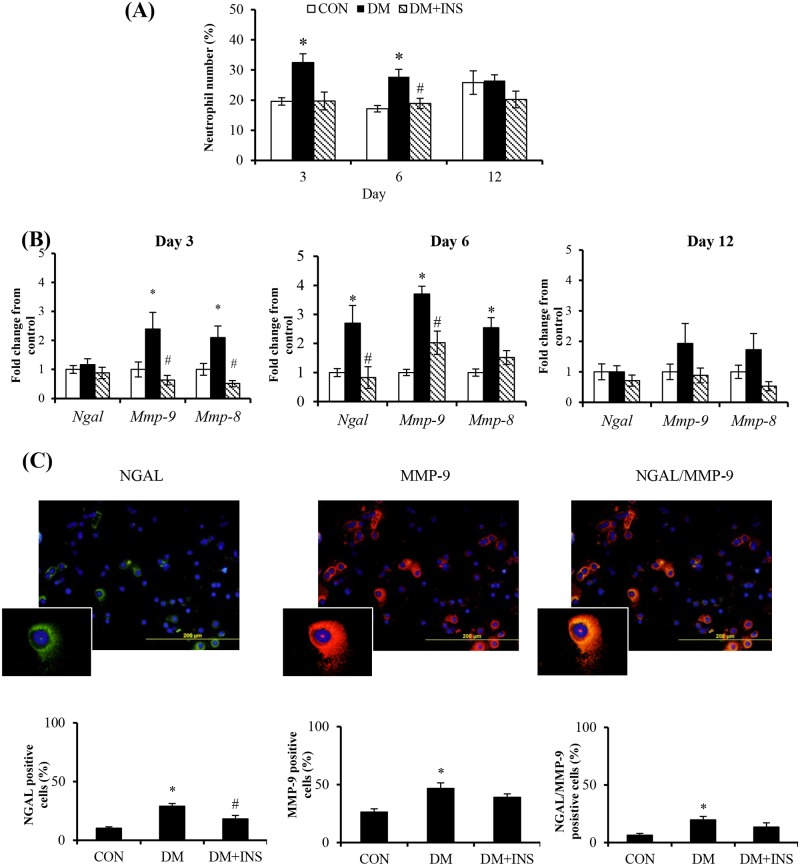
Peripheral blood neutrophil NGAL and MMP-9 at days 3, 6 and 12 post-implant surgery. (A) Neutrophil number as measured by flow cytometry and (B) Gene expression data for NGAL, MMP-9 and MMP-8. (C) Representative and group data for neutrophil NGAL and MMP-9 and their co-localisation at day 6. Results from Control (CON) diabetic (DM) and Insulin treated DM (DM+INS) animals are expressed as Mean ± SEM. * P<0.05 vs. CON, ^#^P <0.05 vs DM

Whether these changes in PVC implant neutrophils also occur in circulating neutrophils from the same animals was next examined. As shown compared with control, diabetes significantly increased circulating neutrophil number (Fig 4A) and neutrophil MMP-9 and MMP-8 mRNA (Fig 4B) at days 3 and 6. Neutrophil NGAL mRNA levels were increased diabetic animals at day 6 (P<0.05). As shown in day 6 representative images and group data (Fig 4C) compared with control, the percentage of NGAL, MMP-9 and NGAL/MMP-9 positive neutrophils was increased in diabetic animals. Across all groups and different to the pattern observed in the PVC implant model more circulating neutrophils were MMP-9 positive (Circulating:25–50% vs Implant: 5–25%). The majority of NGAL positive neutrophils also expressed MMP-9. Insulin treatment prevented the changes in mRNA level as well as the increase in NGAL positive cells but had no effect on the percentage of MMP-9 or combined NGAL/MMP-9 positive cells.

### The effect of diabetes on circulating neutrophil, skin wound and PVC implant TLRs and TNFα

**Fig 5:**
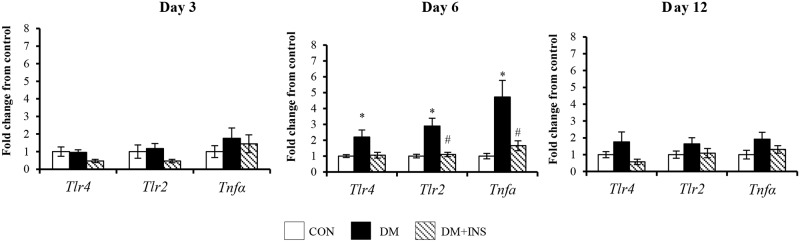
Peripheral blood neutrophil TLR4, TLR2, and TNFα mRNA at days 3, 6 and 12 post-surgery. Results from in Control (CON), Diabetic (DM) and Diabetic + Insulin treated (DM+INS) treated animals are expressed as Mean ± SEM. * P<0.05 vs. CON ^#^P <0.05 vs. DM.

The mRNA levels of TLR2, TLR4 and the pro-inflammatory cytokine, TNFα, were measured in circulating neutrophils, PVC implant cells and skin wound granulation tissue as markers of inflammation. In skin wound granulation tissue and PVC implant cells their expression was not altered by diabetes (Table B S1 File). In contrast, in circulating neutrophils (Fig 5), the expression of TLR4, TLR2, and TNFα was transiently increased at day 6 post wounding (all P<0.05). Insulin treatment from onset of diabetes prevented the changes observed at day 6.

### The effect of glucose on isolated neutrophil NGAL and MMP-9 mRNA

**Fig 6:**
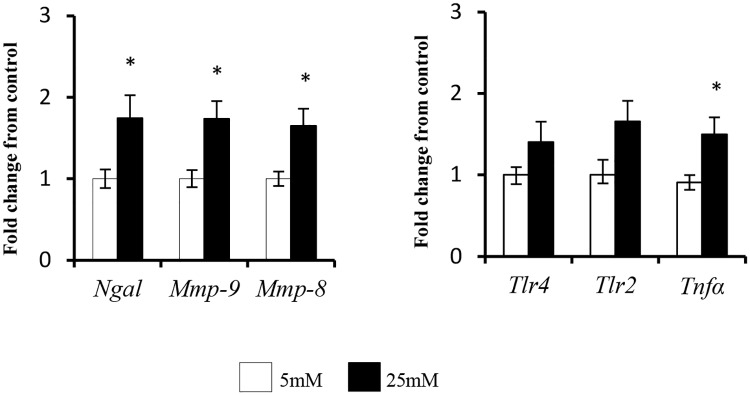
The effect of incubation in high glucose concentration (25mM) on neutrophil (A) NGAL, MMP-9 and MMP-8 mRNA and (B) TLR4, TLR2 and TNFα mRNA. Results are expressed as Mean ± SEM. * P<0.05 vs 5mM glucose control.

Shown in Fig 6, incubation of isolated neutrophils in 25mM glucose for 2 hours significantly increased expression of NGAL, MMP-9, and MMP-8. High glucose also significantly increased TNFα gene expression after 2 hours of incubation and tended to increase neutrophil TLR2 and TLR4. These changes were not due to differences in neutrophil apoptosis as the anti- apoptosis marker Mcl-1 was not altered by culture in the higher glucose concentration (5mM glucose: 100±15% vs 25mM glucose 96±5%). Incubation of neutrophils in mannitol as osmotic control was without effect (data not shown).

## Discussion

Delayed wound healing represents a major health problem and is a common experience for many people with diabetes [22]. Whilst intensive research effort has focussed on some complications of diabetes the pathophysiology of poor wound healing has received less attention. We and others have shown that the concentration and activity of MMP-9 is increased in fluids from poorly healing wounds [9] and can predict delayed healing [11]. In this study we have focussed on MMP-9 expressed by neutrophils and examined the expression and activities of one of its regulators NGAL, in two different wound models. Our results have shown a diabetes related increase in neutrophil number in the circulation as well as in the cellular fraction obtained from the implant. Additionally in these acute inflammatory models, we show an increase in wound MMP-9 activity, increased NGAL mRNA and the formation and persistence of NGAL/MMP-9 complex in the diabetic animals. These changes occur in association with an increase in activation of circulating neutrophils, as identified by increased NGAL and MMP-9 at both the gene and protein level. Moreover insulin treatment from the onset of diabetes can ameliorate some but not all of these changes suggesting that glycaemic control, or at least the anabolic effects of insulin therapy, are important but are not sufficient to improve neutrophil function and wound healing. Additionally our cell culture study suggests that high glucose concentration is in part responsible for these changes.

The published data regarding the effect of diabetes on neutrophil function is conflicting. In peripheral neutrophils from rodents and humans alterations in cytokine production[7], respiratory burst[23], bacterial killing [24] and increased NETosis [3] have been reported but others have failed to detect some of these changes [25]. In this study, in acute wound models, and similar to other studies in excisional wounds well as studies using foreign body implants [17, 26] diabetes delayed healing and inflammatory cell numbers were increased. In our study, we show an increase in neutrophils and this was particularly evident in the PVC implant model where neutrophil number was increased as was the expression of MMP-8 which is highly expressed by neutrophils.

Previous studies in chronic wounds and in poorly healing wounds from diabetic patients have shown high levels of MMP-9 but the source of the MMP-9 and the contribution by neutrophils not been studied [9, 11]. In this study, we used zymography to examine the various forms of MMP-9 in diabetic and control rodent PVC implant fluids. These results show a transient increase in the implant fluid MMP-9 and the NGAL/MMP-9 complex from diabetic animals at day 6. The increase in MMP-9 was due to increased formation of the biologically active 82kDa form of MMP-9 rather than an increase in the proenzyme (92kDa form). Our results show temporal changes in MMP-9 activities and NGAL/MMP-9 complex formation in this model with increased levels of active MMP-9 preceding the formation of the NGAL/MMP-9 complex, the latter which then persisted. Whilst it is not possible to histologically distinguish between the active and the pro form of MMP-9 we also observed at day 6 a similar pattern of change in skin excisional wound MMP-9 and NGAL expression. Interestingly in the PVC implant model when we focussed on neutrophil specific changes, increased neutrophil NGAL preceded any change in neutrophil MMP-9. That there was no concomitant increase in MMP-9 gene expression suggests the observed effect on PVC implant fluid MMP-9 activity occurs via post-translational mechanisms. Additionally the observation of increased formation of the NGAL/MMP-9 heterodimer at days 6 and 12 that can stabilise MMP-9 to prevent it from degradation [27, 28], suggests that in diabetic wounds increased NGAL can act to provide a reservoir of MMP-9, which is available for later release.

In most cell types MMP activities are regulated by specific inhibitors called TIMPs [8]. It is possible that a decrease in TIMP-1 could contribute to increased MMP-9 activities observed in this study. However our previous studies in fluids from an implant model in diabetic baboons [26] and also in unpublished data from rat wound tissue have shown that TIMP-1 is not altered by diabetes. Moreover as neutrophils secrete MMPs in the absence of TIMPs [13] alterations in TIMP would not affect neutrophil formation of the NGAL/MMP-9 complex which we have observed to be elevated in this work.

In diabetic animals the subpopulation of peripheral neutrophils either from the circulation or the wound, which showed peri-nuclear staining for NGAL was more abundant. Additionally at day 6, the time when the most prominent diabetes related changes in PVC implant inflammatory cell neutrophil markers (NGAL and MMP-8) were observed, there was a parallel increase in the expression of these markers of peripheral blood neutrophils. In contrast, at this time point in the peripheral neutrophils but not the wound cells, the gene expression of MMP-9 as well as the inflammatory markers TNFα, TLR2 and TLR4, were all increased. The increased neutrophil expression of TLRs observed in this similar to that seen by Dasu *et al* in circulating monocytes in humans and in db/db mouse models [29]. Why these peripheral blood neutrophil mRNAs are increased but wound expression is unaltered is not known. At the mRNA level differences in cellular composition of neutrophils in the peripheral blood versus a mixture of neutrophils and monocytes in the sponge implant model, is one possible explanation. However this is unlikely as in the implant model neutrophils are two times more abundant than the macrophages. Another possibility is that at day 6 there is an up-regulation of generalised inflammation in the diabetic animal, which leads to increased activation of circulating neutrophils. Together these changes may cause degranulation and removal of a subset of neutrophils from the circulation but the mechanism how this is translated to unaltered change in expression of these mRNAs in the PVC implant cells remains to be further studied. The lack of change in TLRs and TNFα in skin wound granulation tissue was also unexpected and different to previously published work [30]. This lack of effect may be due to the wound model the tissue studied (i.e cells from a sterile implant model vs debrided wound tissue) the species studied or the timing of the study.

In humans the hyperglycaemia of diabetes is known to increase monocyte TLRs [31] but to our knowledge there is no data regarding its effect on neutrophil TLRs. In this rodent study we show in circulating neutrophils but not excisional skin wound or PVC implant cells a transient increase in TLR and TNFα gene expression in association with increased expression of inflammatory markers such as NGAL and MMP-9. This data suggests a diabetes associated alteration in circulating neutrophil phenotype, in response to wounding. Our *ex vivo* studies would suggest that high glucose concentration can contribute to this change, but whether increased responsiveness to circulating damage associated proteins also plays a role and why this pattern is not seen in the cellular wound compartment is as yet not clear.

## Conclusion

This work identifies novel diabetes related differences in circulating and wound tissue neutrophil NGAL and MMP-9. These results show for the first time persistence of the NGAL/MMP-9 complex in wound fluids. As this complex can stabilise MMP-9 this increase suggests a possible mechanism by which MMP-9 levels are maintained at high levels in poorly healing and chronic ulcers. Further as neutrophils are critical for the initiation and progression of wound repair a better understanding of the effect of diabetes on their function at the various phases of wound repair is required. Whether these changes are exacerbated clinically in wounds in people with diabetes where healing is often more prominently delayed and infection risk is high remains to be studied.

## Supporting information

S1 File (DOC)
