# Supplementary material for: Insulin treatment prevents wounding associated changes in tissue and circulating neutrophil MMP-9 and NGAL in diabetic rats
Source: PLoS One. 2017 Feb 9;12(2):e0170951. doi: 10.1371/journal.pone.0170951 (PMC5300126; doi:10.1371/journal.pone.0170951)
Supplement: S1 File — (DOC) [file pone.0170951.s001.doc]

**Supporting Information**

**Table A S1 File. Primers used in this study**

| Target gene  (rat) | Forward primer (5’-3’) | Reverse primer (5’-3’) |
| --- | --- | --- |
| NGAL  (Lipocalin-2) | TCCATCCTCGTCAGGGGCCA | AGTGTCGGCCACTTGCACATCG |
| 36B4 | GTACCATTGAAATCCTGAGCGA | GCCATTGTCAAACACCTGCT |
| MMP-9 | GCTTAGATCATTCTTCAGTGCC | GTTTAGAGCCACGACCATACAG |
| MMP-8 | CAAGACTCCAAGAATTACAACCTG | TCTCTGTAACCATAGTTTGGGT |
| TLR4 | ATTGTTCCTTTCCTGCCTGAG | CTAGGTTCTTGGTTGAATAAGG |
| TLR2 | AGGTCTCCAGGTCAAATCTC | CTTTGTCTTTGCTGTGAGTCC |
| TNFα | GCCTCTTCTCATTCCTGCTC | AAGATGATCTGAGTGTGAGGGT |

**Figure A S1 File**

CON DM DM+INS


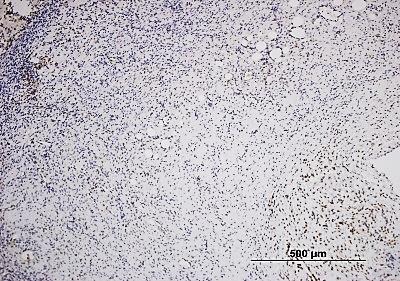

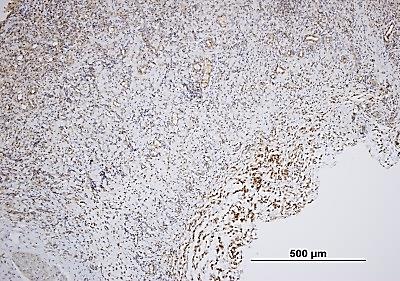

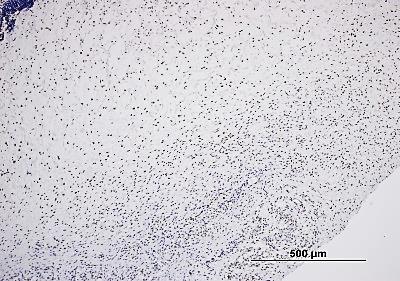

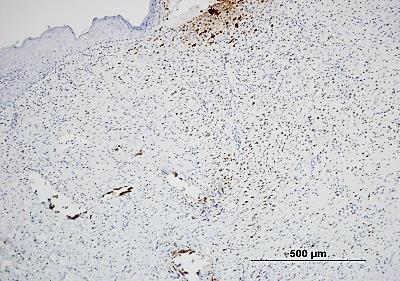

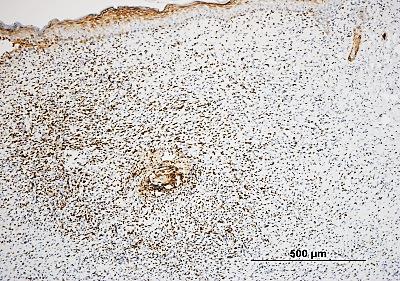

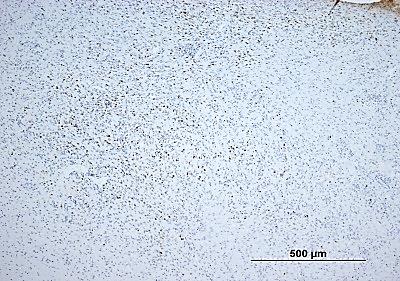


NGAL

MMP-9

MMP-9

Immunohistochemical staining of NGAL and MMP-9 in wound tissue from control (CON), diabetic (DM) and Insulin treated DM animals (DM+INS) at day 6 post wounding. As shown staining intensity for both NGAL and MMP-9 is higher in DM animals and this increase is prevented by insulin treatment

**Figure B S1 File**


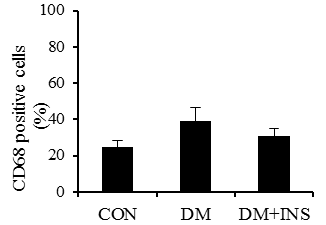

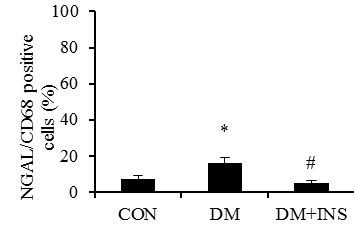

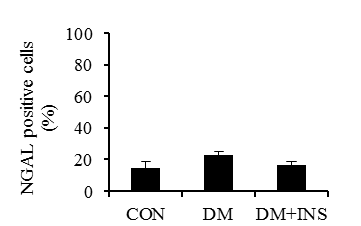


**NGAL NGAL/CD68**

**CD68**


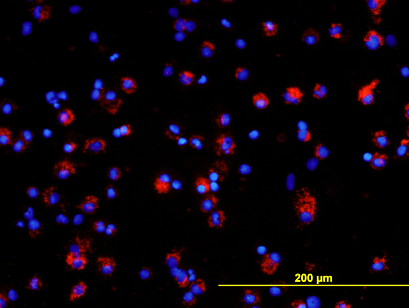

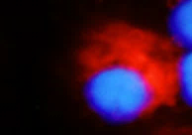

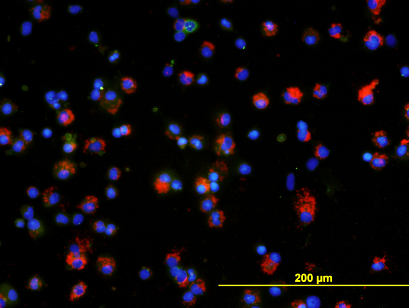

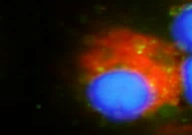

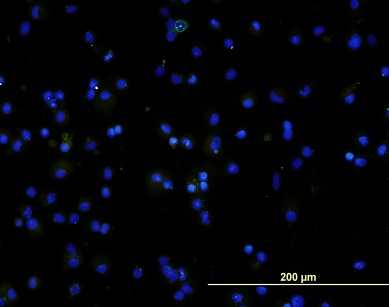

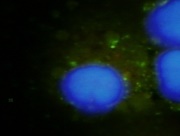


NGAL/CD68 colocalisation in the implant inflammatory cells at day 6. Representative images and group data for macrophage NGAL and CD68. Results from Control (CON) diabetic (DM) and Insulin treated DM (DM+INS) animals are expressed as Mean ± SEM. * P<0.05 vs. CON, ^#^P <0.05 vs DM

**Table B S1 File** The effect of diabetes on wound implant and granulation tissue mRNA levels of TLRs and TNFα

| Wound type | Parameter | Group | | |
| --- | --- | --- | --- | --- |
|  |  | CON | DM | DM+INS |
| Excisional wound | TLR4 | 1.00±0.25 | 1.83±0.27 | 1.36±0.36 |
| Day6 | TLR2 | 1.00±0.28 | 1.57±0.28 | 0.77±0.13 |
| Implant cells | TLR4 | 1.00±0.12 | 0.82±0.10 | 0.67±0.06 |
| Day3 | TLR2 | 1.00±0.11 | 0.88±0.11 | 0.58±0.07 |
|  | TNFα | 1.00±0.16 | 1.01±0.17 | 0.69±0.11 |
| Implant cells | TLR4 | 1.00±0.11 | 1.34±0.10 | 0.89±0.09 |
| Day6 | TLR2 | 1.00±0.09 | 0.82±0.13 | 0.76±0.11 |
|  | TNFα | 1.00±0.11 | 1.09±0.11 | 0.84±0.15 |
| Implant cells | TLR4 | 1.00±0.19 | 0.93±0.23 | 0.90±0.13 |
| Day12 | TLR2 | 1.00±0.20 | 0.85±0.2 | 1.09±0.11 |
|  | TNFα | 1.00±0.19 | 1.37±0.22 | 1.73±0.07* |

Diabetes had no effect on expression of TLR2 and TLR4 or TNFα in wound granulation tissue of the implant inflammatory cells. Interestingly insulin treatment of diabetic animals appeared to increase implant cell TNFα at day 12 post implant, but this change was not observed for other mRNAs measured.
